# Restoration of Autophagic Flux Rescues Oxidative Damage and Mitochondrial Dysfunction to Protect against Intervertebral Disc Degeneration

**DOI:** 10.1155/2019/7810320

**Published:** 2019-12-30

**Authors:** Liang Kang, Qian Xiang, Shengfeng Zhan, Yu Song, Kun Wang, Kangcheng Zhao, Shuai Li, Zengwu Shao, Cao Yang, Yukun Zhang

**Affiliations:** Department of Orthopaedics, Union Hospital, Tongji Medical College, Huazhong University of Science and Technology, Wuhan 430022, China

## Abstract

Oxidative stress-induced mitochondrial dysfunction and nucleus pulposus (NP) cell apoptosis play crucial roles in the development of intervertebral disc degeneration (IDD). Increasing studies have shown that interventions targeting impaired autophagic flux can maintain cellular homeostasis by relieving oxidative damage. Here, we investigated the effect of curcumin (CUR), a known autophagy activator, on IDD *in vitro* and *in vivo*. CUR suppressed *tert*-butyl hydroperoxide- (TBHP-) induced oxidative stress and mitochondrial dysfunction and thereby inhibited human NP cell apoptosis, senescence, and ECM degradation. CUR treatment induced autophagy and enhanced autophagic flux in an AMPK/mTOR/ULK1-dependent manner. Notably, CUR alleviated TBHP-induced interruption of autophagosome-lysosome fusion and impairment of lysosomal function and thus contributed to the restoration of blocked autophagic clearance. These protective effects of CUR in TBHP-stimulated human NP cells resembled the effects produced by the autophagy inducer rapamycin, but the effects were partially eliminated by 3-methyladenine- and compound C-mediated inhibition of autophagy initiation or chloroquine-mediated obstruction of autophagic flux. Lastly, CUR also exerted a protective effect against puncture-induced IDD progression *in vivo*. Our results showed that suppression of excessive ROS production and mitochondrial dysfunction through enhancement of autophagy coupled with restoration of autophagic flux ameliorated TBHP-induced human NP cell apoptosis, senescence, and ECM degradation. Thus, maintenance of the proper functioning of autophagy represents a promising therapeutic strategy for IDD, and CUR might serve as an effective therapeutic agent for IDD.

## 1. Introduction

Lower back pain (LBP), one of the most common musculoskeletal problems in modern society, is responsible for low life quality and a large economic burden [1]. The major cause of LBP is widely acknowledged to be intervertebral disc (IVD) degeneration (IDD), an abnormal and cell-mediated response to progressive failure of the IVD due to aging, genetic factors, and various environmental stressors [2]. Currently, surgery and certain limited forms of conservative treatment, such as good rest and pain relief, are used for IDD, but no effective or constructive drugs are employed in the clinical therapy of IDD [3].

The IVD consists of three interrelated structures: the gelatinous nucleus pulposus (NP), the annulus fibrosus (AF), and the cartilaginous endplate. NP cells play a key role in maintaining the normal structure and physiological function of the IVD by generating the main components of the NP extracellular matrix (ECM), including type II collagen and proteoglycans (mainly aggrecan) [4]. IDD typically occurs when NP cell apoptosis and senescence levels are abnormally elevated and ECM catabolism exceeds anabolism [5]. Oxidative stress has been confirmed to be deeply involved in IDD pathogenesis [6–8]. It refers to the imbalance between the generation of reactive oxygen species (ROS) and the ability of cells to counteract them through antioxidant defense and can damage lipids, proteins, and DNA [9]. The pathological factors related to IDD, such as mechanical loading, nutrition deprivation, and production of inflammatory cytokines, can markedly induce ROS production, leading to oxidative stress and subsequent mitochondrial dysfunction, which further results in the excessive apoptosis, senescence, and ECM degradation of NP cells [6]. Thus, comprehensive investigation into the mechanism underlying uncontrolled ROS production and effective interventions against this might not only increase our knowledge regarding IDD pathogenesis but also suggest appropriate therapeutic strategies for retarding IDD.

Autophagy, which is highly conserved in all eukaryotes, not only is the major intracellular degradation system through which damaged organelles and proteins are degraded in lysosomes but also is an active recycling system that produces materials and energy for the renovation and homeostasis of cells [10]. The term “autophagic flux” reflects the completion of the entire dynamic process of autophagosome formation and then fusion with lysosomes, subsequent degradation of cargo, and release of degradation products [11]. Autophagy disturbances are frequently caused by these mechanisms: inhibition of autophagy initiation, interruption of autophagosome-lysosome fusion, and damage to lysosomal function [12]. Moreover, autophagy dysfunction has been widely reported to be closely related to cell senescence and apoptosis in the progression of several diseases, particularly degenerative diseases such as osteoarthritis [13], IDD [14], neurodegenerative disorders [15], and age-related macular degeneration [16]. Notably, Xu et al. found that treatment of rat NP cells with rapamycin (Rap), a classical autophagy activator, can potently prevent ECM degradation induced by inflammatory factors such as tumor necrosis factor-*α* and interleukin-1*β* [17]. Recently, autophagic flux was also reported to be markedly impaired in rat NP cells under oxidative stress [18]. Therefore, autophagy upregulation coupled with autophagic flux enhancement might represent a promising therapeutic strategy for IDD.

Curcumin (CUR) is an active polyphenol derived from the dried rhizomes of *Curcuma longa*, and CUR has been used for dietary and medicinal purposes for hundreds of years in several parts of the world [19]. Because of its inherent anti-inflammatory, antioxidative stress, and anticatabolic properties, CUR can mitigate the pathogenesis and symptoms of various diseases, including osteoarthritis [20], Alzheimer's disease [21], and atherosclerosis [22]. Moreover, CUR has been recently demonstrated to induce autophagy in diverse tissues, such as cartilage [23], heart [24], and liver [25], but whether the NP is included among such tissues remains unknown. Given the established link between autophagy and oxidative stress and the ability of CUR to promote autophagy, an enhanced understanding of how CUR affects the NP might facilitate the development of clinically safe, orally available therapeutic agents for IDD.

In this study, we investigated the effect of CUR on autophagy activity, as well as its protective effect against apoptosis, senescence, ECM degradation, and mitochondrial dysfunction in NP cells under oxidative stress. We used *tert*-butyl hydroperoxide (TBHP) to induce an oxidative microenvironment because it offers several advantages over hydrogen peroxide (H_2_O_2_), such as high stability and slow release [26]. Furthermore, we assessed the therapeutic potential of CUR by using a rat model of puncture-induced IDD, with our aim being to provide novel insights into the development of effective therapeutic strategies to inhibit IDD progression.

## 2. Material and Methods

### 2.1. Patient Tissue Samples

Lumbar NP specimens were obtained from 8 patients (4 males and 4 females; age range: 15–30 years) with idiopathic scoliosis undergoing deformity correction surgery. The study protocol was approved by the Ethics Committee of Tongji Medical College, Huazhong University of Science and Technology (No. S214), and informed consent was obtained from each donor.

### 2.2. Isolation and Culture of Human NP Cells

Human NP cells were isolated as described previously and cultured [27], at 37°C in a humidified 5% CO_2_ atmosphere, in Dulbecco's modified Eagle medium supplemented with F12 nutrient mixture (Gibco, Grand Island, NY, USA), 15% fetal bovine serum (Gibco), and 1% penicillin/streptomycin (Sigma-Aldrich, St. Louis, MO, USA). At confluence, the cells were digested by trypsinization and passaged for expansion, and cells from the second passage were seeded into experimental plates for all assays.

### 2.3. Cell Culture Treatment Protocols

To determine the effect of CUR on TBHP-treated human NP cells, the cells were pretreated with increasing concentrations (10–25 *μ*M) of CUR for 24 h and then treated with TBHP (50 *μ*M) for 24 h. To evaluate how CUR affects autophagy activity, the human NP cells were either treated for 24 h with different concentrations of CUR (0, 5, 10, 15, 20, and 25 *μ*M) or treated with 25 *μ*M CUR for different times (0, 6, 12, 18, 24, and 30 h). To examine whether CUR promotes autophagic flux, the NP cells were pretreated with or without chloroquine (CQ; 10 *μ*M) and then treated with different concentrations of CUR (10, 15, 20, and 25 *μ*M). To test the role of autophagic flux in the protective effects of CUR pretreatment, cells were pretreated with CQ (10 *μ*M), 3-methyladenine (3-MA; 5 mM), or compound C (10 *μ*M) for 2 h before administration of CUR and TBHP; for the positive control group, cells were treated with Rap (100 nM) for 2 h and then treated with TBHP for 24 h. CUR, TBHP, CQ, 3-MA, compound C, and Rap were purchased from Sigma-Aldrich.

### 2.4. Assessment of Cell Viability and Proliferation

The cytotoxic effect of CUR or TBHP on human NP cells was assessed using the cell counting kit- (CCK-) 8 assay (Dojindo, Japan): cells were resuspended and seeded in 96-well plates, incubated for 24 h, and treated with CUR or TBHP for different times and concentrations. Subsequently, 10 *μ*L of CCK-8 solution was added to each well, and the cells were cultured at 37°C for 4 h, after which the absorbance at 450 nm was measured using a spectrophotometer (BioTek, Winooski, VT, USA).

Cell proliferation was also assessed using a BeyoClick™ EdU-488 Cell Proliferation kit (Beyotime, China). The assessment was performed as the procedure recommended by the manufacturer, and fluorescent images were finally obtained by a fluorescence microscope (Olympus IX71, Tokyo, Japan).

### 2.5. RNA Extraction and Quantitative Real-Time PCR (qRT-PCR)

After treatments of human NP cells in the different groups, total RNA was extracted using a TRIzol reagent (Invitrogen, Carlsbad, CA, USA) and reverse-transcribed using a Transcriptor First Strand cDNA Synthesis Kit (Takara Biotechnology, Otsu, Japan). qRT-PCR was conducted using SYBR Green Kit Master Mix (Applied Biosystems, Foster City, CA, USA), and the products were analyzed using an ABI 7500 Sequencing Detection System, according to the manufacturer's instructions. The primers used in this study are listed in Table 1. Relative expression levels of each gene were calculated using the 2^−*ΔΔ*CT^ method. GAPDH was used as an internal control.

### 2.6. Western Blotting Analysis

After various treatments, the total, cytoplasmic, and mitochondrial proteins from cells were extracted using the corresponding kits, according to the manufacturer's instructions (Beyotime, China). Equal amounts of protein from each sample were separated using 10–12% SDS-PAGE and transferred to PVDF membranes (Millipore, USA), which were blocked and incubated first with specific primary antibodies (1 : 500–1 : 1000) overnight at 4°C and then with the appropriate horseradish peroxidase- (HRP-) labeled secondary antibodies (1 : 2000; Abcam). Protein expression was visualized using enhanced chemiluminescence reagents (Amersham, Piscataway, NJ, USA). Primary antibodies against these molecules were used: cytochrome c (ab133504, Abcam), VDAC1 (sc-32063, Santa Cruz Biotechnology), GAPDH (#5174, Cell Signaling Technology), Bax (ab32503, Abcam), Bcl-2 (ab32124, Abcam), cleaved caspase-3 (#9664, Cell Signaling Technology), cleaved caspase-9 (#9505, Cell Signaling Technology), p16 (#80772, Cell Signaling Technology), type II collagen (ab34712, Abcam), aggrecan (13880-1-AP, Proteintech), matrix metalloproteinase- (MMP-) 3 (#14351, Cell Signaling Technology), MMP-13 (ab39012, Abcam), ADAMTS-4 (ab185722, Abcam), ADAMTS-5 (ab41037, Abcam), LC3 (#12741, Cell Signaling Technology), Beclin-1 (#3495, Cell Signaling Technology), SQSTM1/p62 (ab56416, Abcam), AMPK (#5832, Cell Signaling Technology), phospho- (p-) AMPK (#2535, Cell Signaling Technology), mTOR (#2983, Cell Signaling Technology), p-mTOR (#5536, Cell Signaling Technology), ULK1 (#8054, Cell Signaling Technology), p-ULK1 (#12753, Cell Signaling Technology), and lysosomal-associated membrane protein (LAMP) 2 (27823-1-AP, Proteintech).

### 2.7. Short Interfering (si) RNA Transfection

AMPK-targeting siRNA (si-AMPK) and scrambled siRNA (si-Control) were designed and synthesized by RiboBio (Guangzhou, China). NP cells were transfected with siRNAs by using Lipofectamine 2000 (Invitrogen) according to the manufacturer's instructions. After various treatments, cells were harvested for further experiments.

### 2.8. Flow Cytometry

Human NP cells from each treatment group were harvested, and the apoptosis levels, mitochondrial membrane potential (*ΔΨ*m) changes, and intracellular ROS production were assessed using an Annexin V-APC/7-AAD Apoptosis Detection Kit (Yeasen Biotech, China), the fluorescent probe JC-1 (Beyotime), and the ROS-specific fluorescent probe dihydroethidium (DHE, Beyotime), respectively, as previously described [28]. After labeling, samples were examined using a FACSCalibur flow cytometer (BD Biosciences, USA).

### 2.9. Measurement of Intracellular Malondialdehyde (MDA) Level, Superoxide Dismutase (SOD) Activity, ATP Content, and Mitochondrial Permeability Transition Pore (mPTP) Opening

The MDA level, SOD enzymatic activity, ATP production, and mPTP opening in human NP cells were measured using assay kits for MDA (Beyotime), SOD (Nanjing Jiancheng Bioengineering Institute, China), ATP (Beyotime), and mPTP (Genmed, Shanghai, China), respectively, as per manufacturer instructions.

### 2.10. Immunofluorescence

Immunofluorescence staining of human NP cells was performed as described previously [9]. The cells were incubated overnight at 4°C with primary antibodies against cleaved caspase-3 (1 : 400), type II collagen (1 : 200), MMP-13 (1 : 200), LC3 (1 : 100), or LAMP1 (1 : 200) and then with appropriate secondary antibodies for 2 h at room temperature. Nuclei were stained with DAPI (Beyotime). Fluorescence images were acquired using a fluorescence microscope (Olympus IX71, Tokyo, Japan) and a laser-scanning confocal microscope (ZEISS LSM780, Germany).

### 2.11. Senescence Assessment

Intracellular senescence-associated *β*-galactosidase (SA-*β*-gal) activity was evaluated using an SA-*β*-gal staining kit (Beyotime), according to the manufacturer's instructions. After staining, SA-*β*-gal staining-positive senescent NP cells appeared blue under a light microscope. SA-*β*-gal activity was expressed as the percentage of SA-*β*-gal staining-positive NP cells to total NP cells.

### 2.12. mRFP-GFP-LC3 Assay

Human NP cells were infected with mRFP-GFP-LC3 adenoviral vectors (Hanbio Technology, Shanghai, China) to evaluate the effect of TBHP and CUR on autophagic flux. The principle of the assay is based on difference in pH stability of the green and red fluorescent proteins: whereas the fluorescence signal of GFP is quenched under the acidic condition inside the lysosome, the mRFP fluorescence signal does not change markedly under the acidic condition; therefore, autophagosomes appear as yellow puncta (RFP+GFP+), but autolysosomes appear as red puncta (RFP+GFP−). When autophagic flux is increased, both yellow and red puncta are increased in cells, whereas when autophagic flux is blocked, yellow puncta are increased but red puncta are not altered. The mRFP and GFP puncta in each treatment group were detected using a laser-scanning confocal microscope (ZEISS LSM780).

### 2.13. Measurement of Cathepsin B (CTSB) Activity, Cathepsin D (CTSD) Activity, and Lysosomal pH Changes

CTSB and CTSD activities were measured using specific fluorometric assay kits for the two enzymes (BioVision), as described previously [29]. Changes in lysosomal pH were determined using LysoSensor Green DND-189 (Yeasen), as reported; the reagent exhibits a pH-dependent increase in fluorescence intensity in an acidic environment. The cells were examined and imaged using a fluorescence microscope.

### 2.14. Rat IDD Model

The animal experimental protocols were approved by the Animal Experimentation Committee of Huazhong University of Science and Technology. Sprague-Dawley rats (3 months old) were obtained from the Laboratory Animal Center of Huazhong University of Science and Technology (Wuhan, China) and divided randomly into three groups (control, IDD, and CUR groups); the animals in the IDD and CUR groups underwent IVD puncture surgery, which was performed using a needle (27G) on the experimental-level rat tail disc (Co7/8) according to the surgical methods reported by Chen et al. [30]. After surgery, the CUR group was injected intraperitoneally with 100 mg/kg CUR and the IDD group was injected with saline twice weekly for one month. The animals were monitored daily to ensure their well-being, and all animals were allowed free unrestricted weight bearing and activity.

### 2.15. Magnetic Resonance Imaging (MRI) Evaluation

MRI was performed on all rats by using a BioSpec MRI (Bruker, 7.0 T/20 cm). The parameters of T2-weighted imaging were those referred to in a previous study [27]. The Pfirrmann classification system [31] was used to assess the grades of IVD degeneration.

### 2.16. Western Blotting Analysis, H_2_O_2_ Content Measurement, Histological Assessment, and Immunohistochemical Staining in Animal Models

After MRI examination, disc tissues were harvested for further analyses. The specimens were fixed in formaldehyde, decalcified, dehydrated, embedded in paraffin, and sectioned at a thickness of 5 *μ*m. Sections were stained with hematoxylin-eosin (HE) and safranin O-fast green (SO), and the histological scores were calculated based on the method previously described [32] to quantify the histological results. Immunohistochemistry was performed as described [14]. For immunohistochemical analysis, sections were incubated with primary antibodies against type II collagen, MMP-13, LC3, p62, and cleaved caspase-3 at 4°C overnight, after which the sections were incubated with appropriate HRP-conjugated secondary antibodies and counterstained with hematoxylin. Western blotting was used to detect the expression of type II collagen, aggrecan, MMP-13, ADAMTS-4, ADAMTS-5, LC3, p62, and cleaved caspase-3 in the disc tissues in the same steps as above. Lastly, the content of H_2_O_2_ in the disc tissues was assessed using a Hydrogen Peroxide assay kit (Nanjing Jiancheng Bioengineering Institute) according to the manufacturer's instructions.

### 2.17. Statistical Analysis

Data are presented as means ± standard deviation (SD) of at least three independent experiments and were analyzed using SPSS v.18.0 software (SPSS, Chicago, IL, USA). Differences between groups were evaluated using Student's *t*-test or one-way analysis of variance (ANOVA) followed by Tukey's test. *P* < 0.05 was considered statistically significant.

## 3. Results

### 3.1. Effect of CUR on Human NP Cell Viability

**Figure 1:**
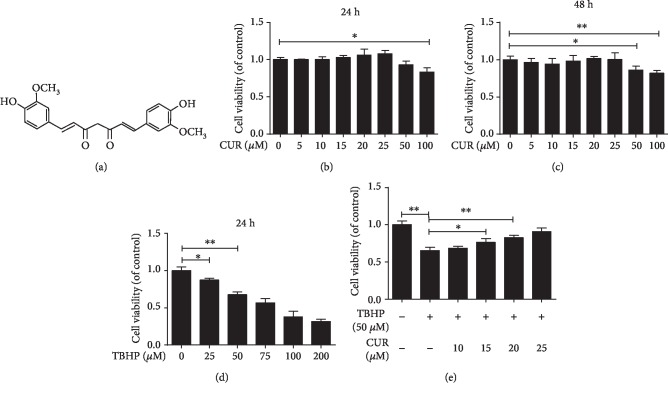
CUR attenuates TBHP-induced death in human NP cells. (a) The chemical structure of CUR. (b, c) CCK-8 assay was used to determine the cytotoxic effects of CUR on human NP cells by treating the cells with various concentrations of CUR for durations of 24 h and 48 h. (d) The viability of the human NP cells treated with different concentrations of TBHP for 24 h was determined by the CCK-8 assay. (e) The results of the CCK-8 assay performed on CUR-pretreated human NP cells induced by TBHP. Data are represented as the mean ± SD. ^∗∗^*P* < 0.01, ^∗^*P* < 0.05, *n* = 3.

The chemical structure of CUR is shown in Figure 1(a). The results of the CCK-8 assay showed that at ≤25 *μ*M, CUR was not cytotoxic to human NP cells for 24 and 48 h (Figures 1(b) and 1(c)). Treatment with TBHP for 24 h reduced the viability of human NP cells in a dose-dependent manner, and 50 *μ*M TBHP was selected for stimulating cells in subsequent experiments (Figure 1(d)). Notably, CUR pretreatment attenuated the cell death induced by TBHP (Figure 1(e)).

### 3.2. Effect of CUR on Senescence and Apoptosis in TBHP-Treated Human NP Cells

**Figure 2:**
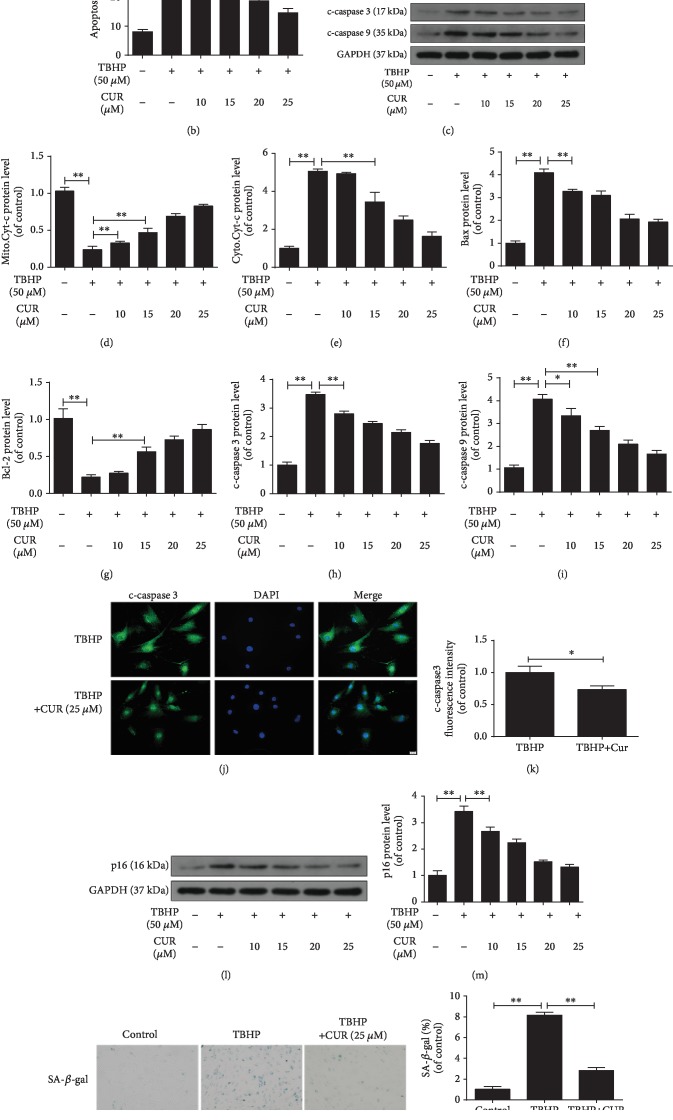
CUR treatment inhibits TBHP-induced apoptosis and senescence in human NP cells. (a, b) Annexin V-APC/7-AAD staining results showing the rate of apoptosis in human NP cells. (c–i) The protein levels of mitochondrial Cyt-c, cytoplasmic Cyt-c, Bax, Bcl-2, cleaved caspase-3, and cleaved caspase-9 in human NP cells were measured by western blotting. (j, k) Immunofluorescence staining of cleaved caspase-3 in human NP cells. Scale bar: 20 *μ*m. (l–o) After the indicated treatment, cells were washed and then cultured under normal conditions for the indicated time. (l, m) After 24 h, the level of p16 protein in the human NP cells was measured by western blotting. (n, o) After 3 days, the SA-*β*-gal staining assay was performed in the human NP cells. Scale bar: 100 *μ*m. (p, q) Cell proliferation was detected by EdU staining under a fluorescence microscope, and the positive cells were quantitated. Scale bar: 50 *μ*m. GAPDH was used as an internal control. Data are represented as the mean ± SD. ^∗∗^*P* < 0.01, ^∗^*P* < 0.05, *n* = 3.

Next, the effect of CUR on apoptosis of human NP cells exposed to TBHP was assessed through Annexin V-APC/7-AAD staining. TBHP treatment significantly increased the incidence of NP cell apoptosis, whereas CUR pretreatment rescued this TBHP-induced increase of apoptosis (Figures 2(a) and 2(b)). Moreover, we used western blotting to measure mitochondrial pathway apoptosis-related proteins (Bcl-2, Bax, cleaved caspase-3, cleaved caspase-9, and cytochrome c) in human NP cells; this is because NP cell apoptosis induced by oxidative stress is reported to be mediated at least partially through activation of the mitochondrial pathway. TBHP treatment increased the levels of Bax, cleaved caspase-3, cleaved caspase-9, and cytoplasmic cytochrome c and decreased the levels of Bcl-2 and mitochondrial cytochrome c, but CUR pretreatment attenuated these changes (Figure 2(c)–2(i)). Immunofluorescence staining further revealed that the level of cleaved caspase-3 was lower in the TBHP/CUR cotreatment group than in the group treated with TBHP alone (Figures 2(j) and 2(k)). Lastly, the results of senescence detection in human NP cells coincided with the apoptosis results, as shown by western blotting for p16 (a classical senescence marker) (Figures 2(l) and 2(m)), SA-*β*-gal staining (Figures 2(n) and 2(o)), and EdU incorporation (Figures 2(p) and 2(q)). Together, these results indicate that CUR exerts antiapoptosis and antisenescence effects in TBHP-treated human NP cells.

### 3.3. Effect of CUR on TBHP-Induced ECM Degradation in Human NP Cells

**Figure 3:**
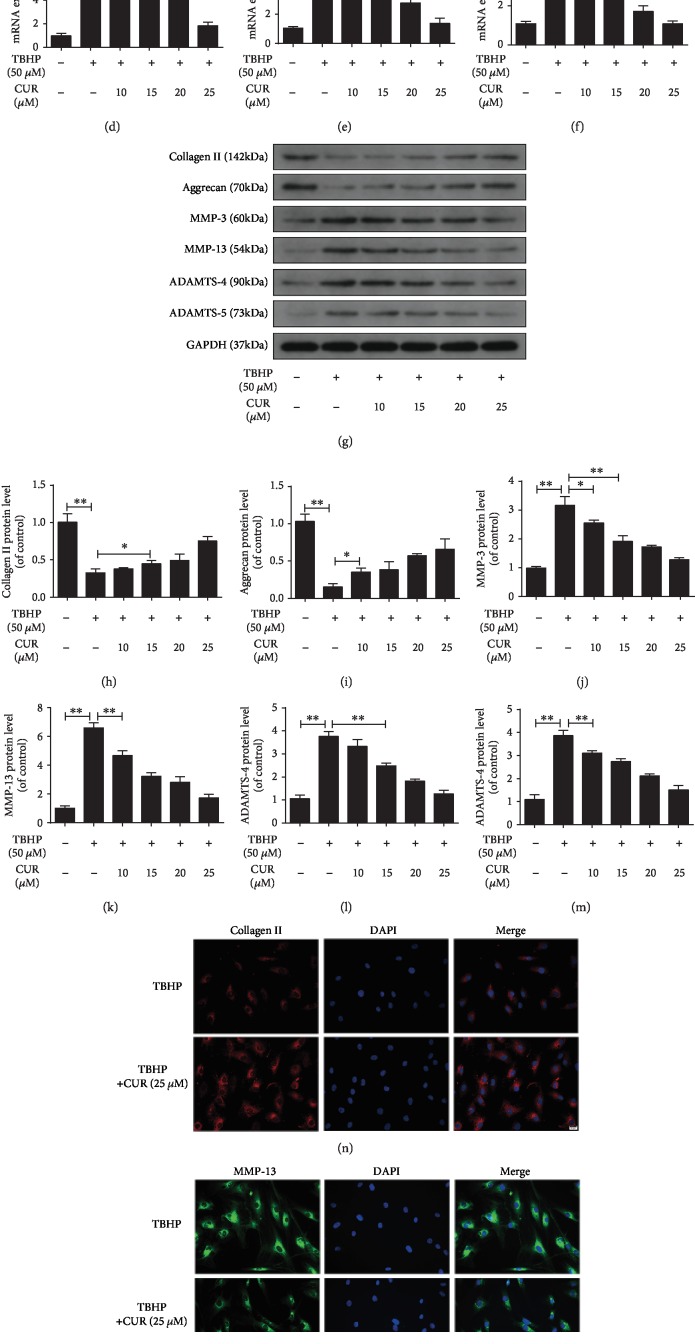
CUR treatment alleviates TBHP-induced degradation of the ECM in the human NP cells. (a–f) The mRNA expression levels of type II collagen, aggrecan, MMP-3, MMP-13, ADAMTS-4, and ADAMTS-5 in the human NP cells were measured by qRT-PCR. (g–m) The protein levels of type II collagen, aggrecan, MMP-3, MMP-13, ADAMTS-4, and ADAMTS-5 in the human NP cells were measured by western blotting. (n–q) Immunofluorescence staining of type II collagen and MMP-13 in the human NP cells. Scale bar: 20 *μ*m. GAPDH was used as an internal control. Data are represented as the mean ± SD. ^∗∗^*P* < 0.01, ^∗^*P* < 0.05, *n* = 3.

The imbalance between ECM anabolism and catabolism in NP cells and the consequent ECM degradation are regarded as the main feature of IDD. Therefore, we measured the mRNA and protein levels of ECM anabolism markers (type II collagen and aggrecan) and ECM catabolism markers (MMP-3, MMP-13, ADAMTS-4, and ADAMTS-5) in human NP cells under TBHP stimulation with or without CUR pretreatment. TBHP treatment decreased type II collagen and aggrecan and increased MMP-3, MMP-13, ADAMTS-4, and ADAMTS-5 mRNA levels (Figures 3(a)–3(f)) and protein levels (Figures 3(g)–3(m)), and these TBHP-induced alterations were partially reversed by CUR pretreatment. Moreover, the results of immunofluorescence staining for type II collagen and MMP-13 agreed with the western blotting results (Figures 3(n)–3(q)).

### 3.4. Effect of CUR on TBHP-Induced Oxidative Stress and Mitochondrial Dysfunction in Human NP Cells

**Figure 4:**
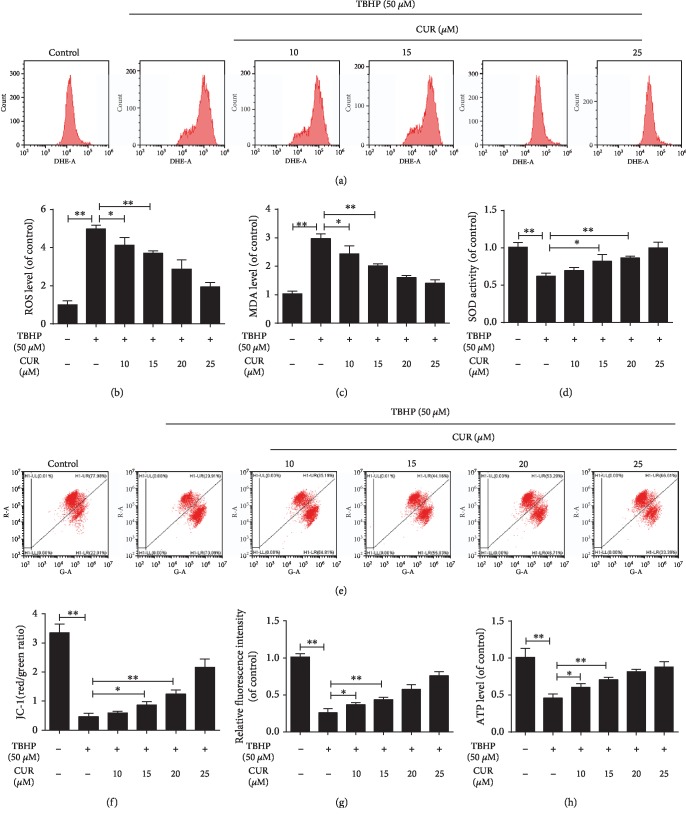
CUR treatment alleviates TBHP-mediated oxidative stress and mitochondrial dysfunction in the human NP cells. (a, b) The ROS levels in the human NP cells were detected using the fluorescent probe DHE and measured by flow cytometry. (c, d) Intracellular MDA levels and SOD activity in the human NP cells. (e, f) Mitochondrial membrane potential was detected by JC-1 staining and measured by flow cytometry. (g) An assessment of mPTP opening in the human NP cells. (h) Intracellular ATP levels in the human NP cells. Data are represented as the mean ± SD. ^∗∗^*P* < 0.01, ^∗^*P* < 0.05, *n* = 3.

Oxidative stress and subsequent mitochondrial dysfunction induced by excessive ROS generation were previously confirmed to play a vital role in IDD progression [9]. Here, intracellular ROS levels were detected and measured using the fluorescent probe DHE and flow cytometry: TBHP treatment markedly induced ROS production, and this was partially inhibited by CUR pretreatment (Figures 4(a) and 4(b)). We also examined the level of MDA, a product of lipid peroxidation that is widely regarded as a marker of oxidative stress-induced cell injury; in TBHP-treated human NP cells, the MDA level was higher than that in control NP cells, and CUR pretreatment partially decreased the TBHP-induced MDA production (Figure 4(c)). We next examined SOD, a key intracellular antioxidant that catalyzes the conversion of superoxide anion (O_2_^–^) to H_2_O_2_, which is then further reduced to H_2_O and O_2_; SOD activity in human NP cells was significantly downregulated after TBHP stimulation, and this effect was largely prevented by pretreatment with CUR (Figure 4(d)). Mitochondrial dysfunction caused by oxidative stress is characterized by the collapse of *ΔΨ*m, increase of mPTP opening, and decrease of ATP production. TBHP treatment led to the loss of *ΔΨ*m in human NP cells, which was indicated by a decrease in the ratio of JC-1 red fluorescence (JC-1 aggregates) to green fluorescence (JC-1 monomers), and, as expected, CUR pretreatment alleviated the TBHP-induced loss of *ΔΨ*m (Figures 4(e) and 4(f)). In addition, CUR pretreatment prevented the prolonged mPTP opening induced by TBHP stimulation (Figure 4(g)). TBHP reduced the ATP level in human NP cells, and this was partially restored by pretreatment with CUR (Figure 4(h)). These results suggest that CUR alleviated TBHP-mediated oxidative stress and mitochondrial impairment in human NP cells.

### 3.5. Effect of CUR on Autophagy in Human NP Cells

**Figure 5:**
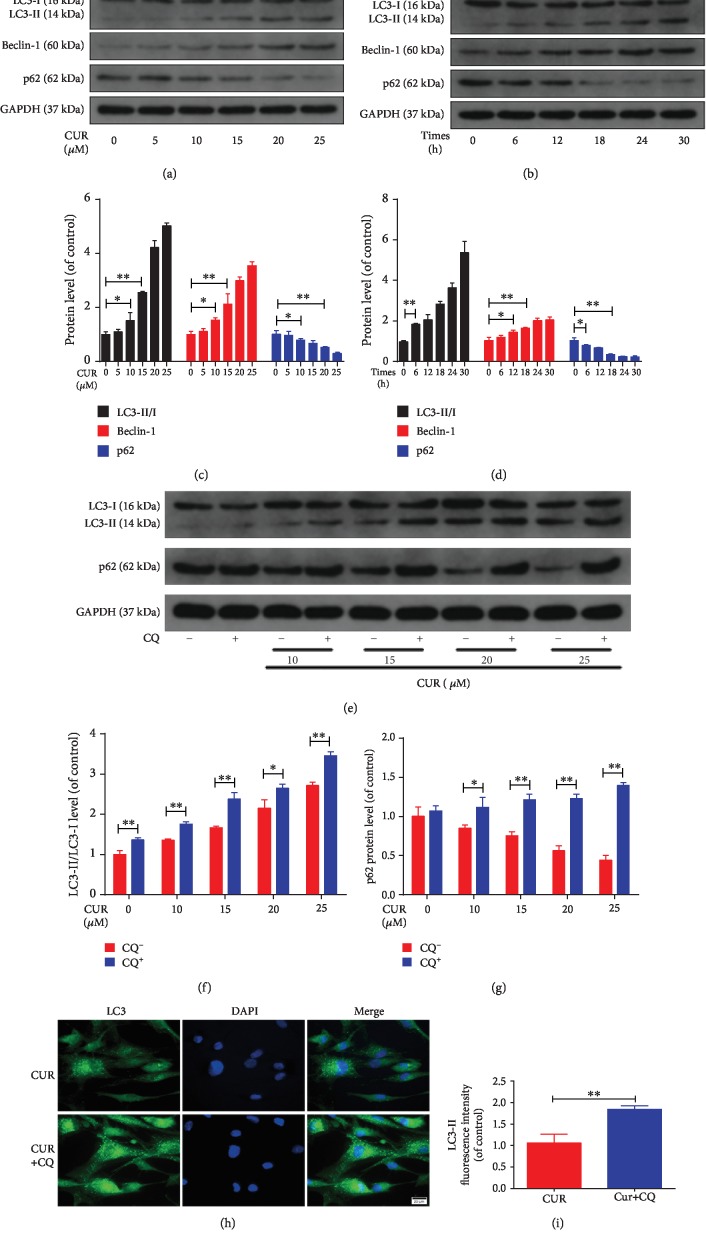
CUR treatment induces autophagy and promotes autophagic flux in the human NP cells. (a–d) The protein levels of LC3, Beclin-1, and p62 in the human NP cells were measured by western blotting. The human NP cells were treated with different concentrations of CUR (0, 5, 10, 15, 20, and 25 *μ*M) for 24 h or treated with 25 *μ*M CUR for different times (0, 6, 12, 18, 24, and 30 h). (e–g) The protein levels of LC3 and p62 in the human NP cells that were pretreated with or without CQ (10 *μ*M) for 2 h and then treated with different concentrations of CUR for 24 h. (h, i) Immunofluorescence staining of LC3 in the human NP cells. Scale bar: 20 *μ*m. GAPDH was used as an internal control. Data are represented as the mean ± SD. ^∗∗^*P* < 0.01, ^∗^*P* < 0.05, *n* = 3.

As indicators of autophagy, LC3-I to LC3-II conversion and Beclin-1 expression were assessed using western blotting. Moreover, we evaluated the levels of p62, a specific substrate degraded in autolysosomes that is used to investigate the status of autophagic flux. The results showed a dose- and time-dependent increase in the LC3-II/I ratio, Beclin-1 expression, and p62 degradation in CUR-treated human NP cells (Figures 5(a)–5(d)). Autophagic flux is a dynamic process involving the formation of autophagosomes, the transport of autophagic substrates to lysosomes, and their subsequent degradation. Because either an elevation of autophagic flux or a reduction of the exhaustion of autophagic vesicles could have resulted in the increased LC3-II levels, we sought to discriminate between these two possibilities and further analyze the role of CUR in autophagic flux: we monitored the LC3-II levels in human NP cells treated with CUR alone or in conjunction with the autophagic flux inhibitor CQ, which suppresses lysosomal acidification and impairs the fusion of autophagosomes with lysosomes. Western blotting analysis revealed that CQ treatment enhanced the level of p62 and the LC3-II/I ratio in CUR-stimulated human NP cells (Figures 5(e)–5(g)), and this was further confirmed through immunofluorescence analysis (Figures 5(h) and 5(i)). Collectively, these results indicate that CUR induces autophagy and promotes autophagic flux in human NP cells.

### 3.6. Effect of AMPK/mTOR/ULK1 Pathway on CUR-Mediated Activation of Autophagy in Human NP Cells

**Figure 6:**
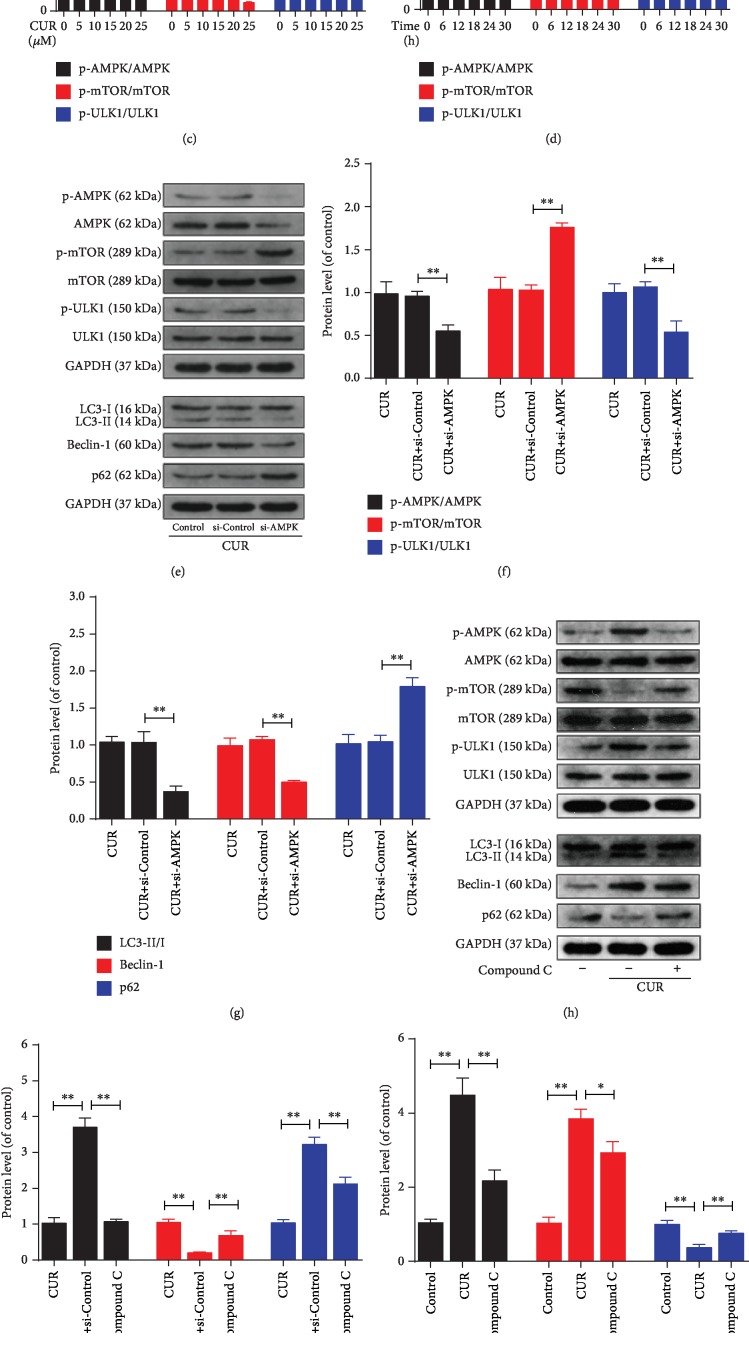
AMPK/mTOR/ULK1 pathway is involved in CUR-triggered activation of autophagy in the human NP cells. (a–d) The levels of AMPK, p-AMPK, mTOR, p-mTOR, ULK1, and p-ULK1 proteins in the human NP cells were measured by western blotting. (e–j) The levels of AMPK, p-AMPK, mTOR, p-mTOR, ULK1, p-ULK1, LC3, Beclin-1, and p62 proteins in the human NP cells that were treated as indicated. CC refers to compound C. (k, l) Immunofluorescence staining of LC3 in human NP cells. Scale bar: 20 *μ*m. GAPDH was used as an internal control. Data are represented as the mean ± SD. ^∗∗^*P* < 0.01, ^∗^*P* < 0.05, *n* = 3.

To identify the signaling pathway associated with autophagy activation in CUR-treated human NP cells, we performed western blotting to assess the levels of AMPK, p-AMPK, mTOR, p-mTOR, ULK1, and p-ULK1 (Figures 6(a)–6(d)): CUR increased the phosphorylation of AMPK and ULK1 and decreased the phosphorylation of mTOR in a dose- and time-dependent manner. Next, human NP cells were transfected with si-AMPK or treated with compound C, a specific AMPK inhibitor, before CUR stimulation. Western blotting results showed that in human NP cells, both compound C and si-AMPK inhibited the CUR-induced upregulation of AMPK and ULK1 phosphorylation and downregulation of mTOR phosphorylation, as well as the CUR-stimulated increase of the LC3-II/I ratio, Beclin-1 expression, and p62 degradation (Figured 6(e)–6(j)). Immunofluorescence staining results also indicated that CUR elevated the LC3-II level and that this could be prevented by compound C pretreatment (Figures 6(k) and 6(l)). These data suggest that the AMPK/mTOR/ULK1 pathway was involved in CUR-triggered autophagy activation in human NP cells.

### 3.7. Effect of CUR on TBHP-Induced Blockage of Autophagic Flux in Human NP Cells

**Figure 7:**
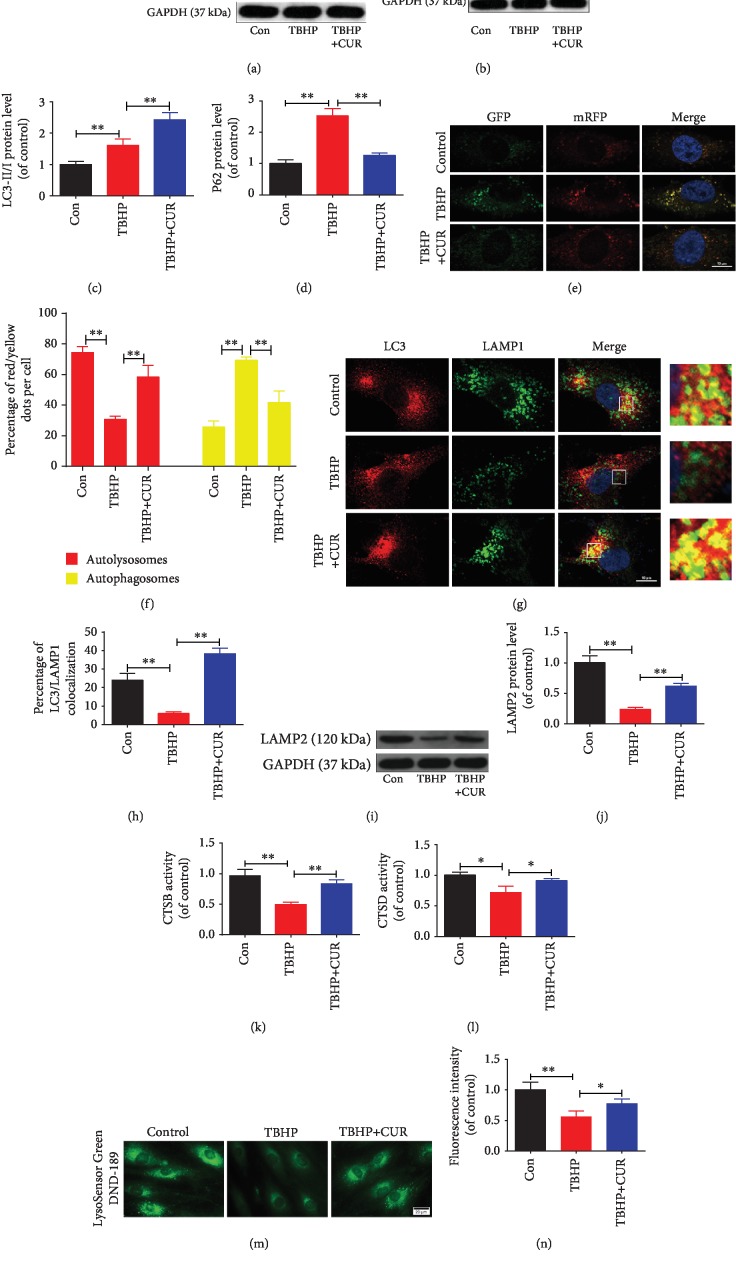
CUR treatment restores defective autophagic flux in the human NP cells subjected to TBHP. (a–d) The levels of LC3 and p62 proteins in the human NP cells were measured by western blotting. (e, f) Representative images of the human NP cells expressing mRFP-GFP-LC3 were obtained by confocal microscopy. Scale bar: 10 *μ*m. (g, h) The colocalization of LC3 and LAMP1 was examined by confocal microscopy. Scale bar: 10 *μ*m. (i, j) LAMP2 protein level in the human NP cells was evaluated by western blotting. (k, l) The activity of CTSB and CTSD in the human NP cells. (m, n) Lysosomal pH of human NP cells was determined by LysoSensor Green DND-189 staining. Scale bar: 20 *μ*m. GAPDH was used as an internal control. Data are represented as the mean ± SD. ^∗∗^*P* < 0.01, ^∗^*P* < 0.05, *n* = 3.

Changes in autophagic flux were evaluated in human NP cells treated with CUR and TBHP. First, western blotting results showed that TBHP treatment enhanced the LC3-II/I ratio and p62 protein level, which suggested impairment of autophagic flux under TBHP exposure, and, as expected, CUR pretreatment decreased TBHP-triggered p62 accumulation and increased the LC3-II/I ratio (Figures 7(a)–7(d)). Next, we transfected a tandem mRFP-GFP-LC3 construct into human NP cells to directly monitor the status of autophagic flux. The acidic lysosomal environment efficiently quenches green fluorescence but not red fluorescence, and this leads to the generation of only red fluorescence in autolysosomes but of both red and green fluorescence (merged as yellow puncta) in autophagosomes. As compared with the control group, the TBHP group showed an increase in yellow puncta but not red puncta, which suggested that autophagic flux was inhibited in these cells; however, in the TBHP/CUR cotreatment group, more red puncta and fewer yellow puncta were detected than in the TBHP group (Figures 7(e) and 7(f)). Together, these data indicate that CUR restores the defective autophagic flux in human NP cells exposed to TBHP.

### 3.8. Effect of CUR on Interruption of Autophagosome-Lysosome Fusion and Impairment of Lysosomal Function in Human NP Cells Stimulated with TBHP

The fusion of the autophagosomes with lysosomes is a critical step in maintaining an intact autophagic flux. When we performed double-immunofluorescence staining for LAMP1 (one of the major protein components of the lysosomal membrane) and LC3, we observed that the colocalization of LC3 with LAMP1 was decreased in human NP cells under TBHP stimulation as compared with that in untreated cells; notably, CUR pretreatment rescued this decrease in colocalization, which demonstrated CUR-mediated reversal of TBHP-inhibited autophagosome-lysosome fusion (Figures 7(g) and 7(h)).

The autophagic degradation and turnover of substrates packaged into autolysosomes is the final step of autophagy. A blockade of autophagic flux has been demonstrated to result from impaired lysosomal function, which is indicated by, for example, the presence of aberrant levels of lysosomal membrane proteins, reduction in the activity of lysosomal enzymes, or decrease in lysosomal acidification. Thus, we evaluated whether the protective effect of CUR against TBHP-impaired autophagic flux was associated with the rescue of lysosomal dysfunction in human NP cells. LAMP2, a lysosomal membrane glycoprotein, is critical for autophagosome-lysosome fusion and autophagic clearance. Western blotting revealed that TBHP stimulation inhibited LAMP2 expression in human NP cells, but pretreatment with CUR partially prevented this inhibitory effect (Figures 7(i) and 7(j)). Second, to evaluate the degradation ability of lysosomes in human NP cells, we measured the activity of CTSB and CTSD, which are key lysosomal proteases: CUR pretreatment alleviated TBHP-induced decrease in the activity levels of CTSB and CTSD (Figures 7(k) and 7(l)). Third, considering that low lysosomal pH is essential for lysosomal enzyme activity, we assessed lysosomal pH by means of LysoSensor Green DND-189 staining; the green fluorescence intensity of this probe increases with the acidification of organelles in a pH-dependent manner. We found that TBHP-treated human NP cells exhibited an elevated lysosomal pH as compared to the control, and CUR pretreatment partially suppressed this lysosomal alkalinization induced by TBHP (Figures 7(m) and 7(n)). Collectively, these results demonstrated that impairment of autophagosome-lysosome fusion and lysosomal function in human NP cells exposed to TBHP can be relieved by CUR.

### 3.9. Role of Autophagic Flux Restoration in CUR Protective Effects on Human NP Cells Treated with TBHP

**Figure 8:**
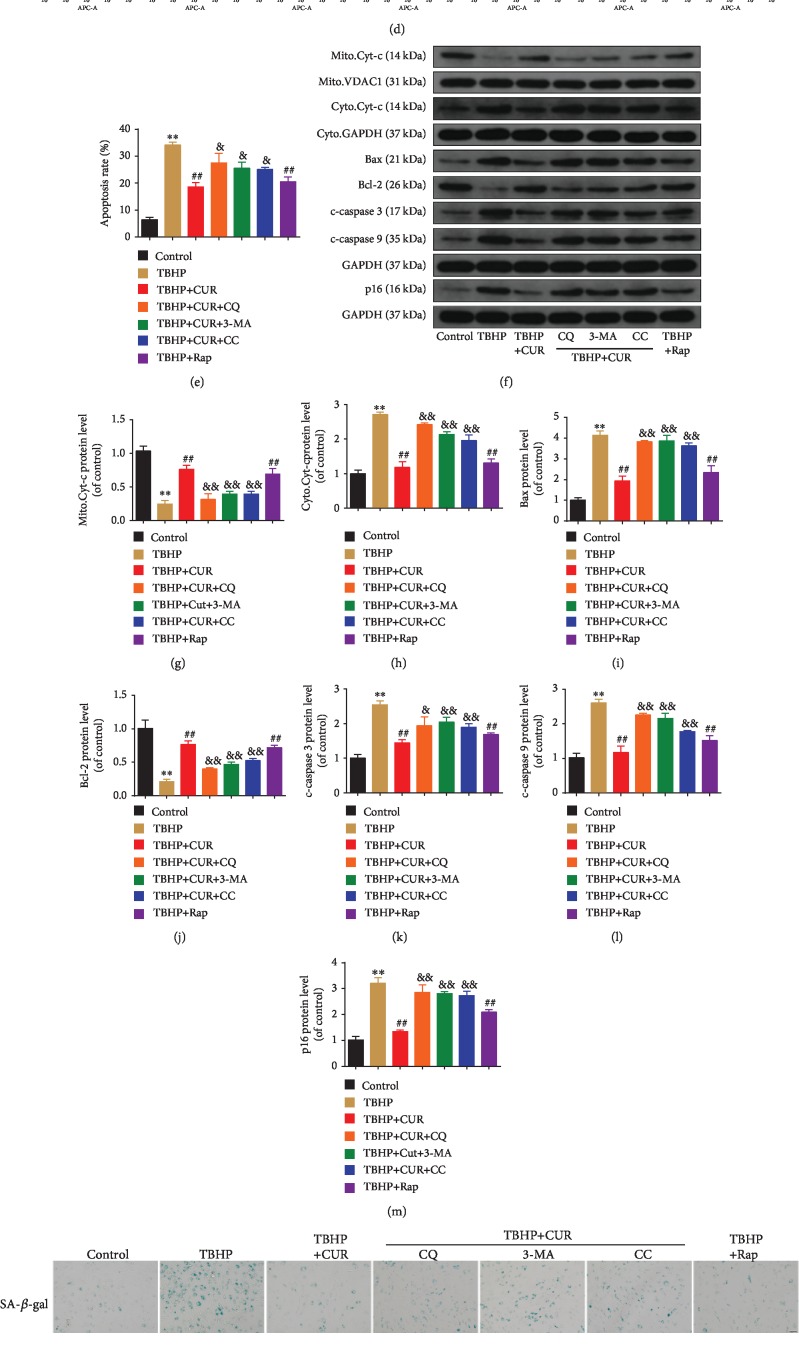
CUR treatment inhibits TBHP-induced apoptosis and senescence in the human NP cells by facilitating autophagic flux. (a–c) The protein levels of LC3and p62 in the human NP cells were measured by western blotting. (d, e) Annexin V-APC/7-AAD staining results showing the apoptosis rate of the human NP cells. (f–l) The expression of mitochondrial Cyt-c, cytoplasmic Cyt-c, Bax, Bcl-2, cleaved caspase-3, and cleaved caspase-9 proteins was measured by western blotting. (f, m–o) Cells were treated as indicated in the legend of Figures 2(l)–2(o). (f, m) The expression of p16 protein was measured by western blotting. (n, o) SA-*β*-gal staining assay was performed in the human NP cells. Scale bar: 100 *μ*m. GAPDH was used as an internal control. Data are represented as the mean ± SD. ^∗∗^*P* < 0.01 versus control group. ^##^*P* < 0.01 versus TBHP group. ^&&^*P* < 0.01, ^&^*P* < 0.05 versus TBHP+CUR group, *n* = 3.

**Figure 9:**
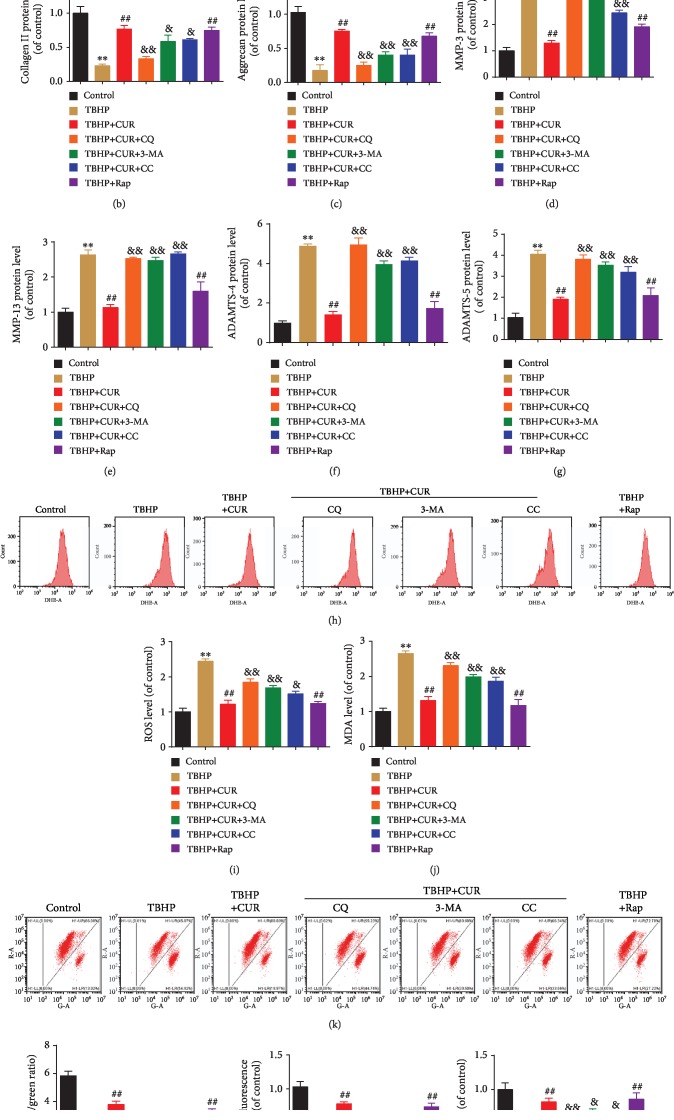
CUR treatment inhibits TBHP-induced ECM degradation, oxidative stresses, and mitochondrial dysfunction by facilitating autophagic flux. (a–g) The protein levels of type II collagen, aggrecan, MMP-3, MMP-13, ADAMTS-4, and ADAMTS-5 in the human NP cells were measured by western blotting. (h, i) The ROS levels were detected using the fluorescent probe DHE and measured by flow cytometry. (j) Intracellular MDA levels in the human NP cells. (k, l) Mitochondrial membrane potential was detected by JC-1 staining and measured by flow cytometry. (m) An assessment of mPTP opening in the human NP cells. (n) Intracellular ATP levels in the human NP cells. GAPDH was used as an internal control. Data are represented as the mean ± SD. ^∗∗^*P* < 0.01 versus control group. ^##^*P* < 0.01 versus TBHP group. ^&&^*P* < 0.01, ^&^*P* < 0.05 versus TBHP+CUR group, *n* = 3.

To investigate the potential contribution of improved autophagic flux to CUR-mediated cytoprotection, human NP cells were treated with the recognized autophagy inhibitors CQ and 3-MA or the AMPK inhibitor compound C and then exposed to CUR and TBHP. Moreover, a classical autophagy inducer, Rap, was used to enhance autophagic flux in TBHP-treated human NP cells. As expected, western blotting for LC3 and p62 showed elevated autophagy under Rap pretreatment as compared with TBHP treatment alone (Figures 8(a)–8(c)). By contrast, pretreatment with either compound C or 3-MA decreased the LC3-II/I ratio and p62 degradation in human NP cells treated with TBHP and CUR, which indicated autophagy inhibition (Figures 8(a)–8(c)). Pretreatment with CQ effectively blocked autophagic flux, as reflected by the increase in the LC3-II/I ratio and p62 level relative to the corresponding ratio and level in the TBHP/CUR cotreatment group (Figures 8(a)–8(c)). Next, we found that when CUR was added to the medium of TBHP-treated human NP cells, TBHP-induced apoptosis and senescence were suppressed, as indicated by the results of flow cytometry (Figures 8(d) and 8(e)), SA-*β*-gal staining (Figures 8(n) and 8(o)), and western blotting analysis of p16, Bcl-2, Bax, cleaved caspase-3, cleaved caspase-9, and cytochrome c (Figures 8(f)–8(m)), which agreed with the results obtained after Rap pretreatment. However, the preventive effects of CUR on TBHP-induced apoptosis and senescence were blunted by CQ, 3-MA, and compound C (Figures 8(d)–8(o)). Moreover, type II collagen and aggrecan levels were lower and MMP-3, MMP-13, ADAMTS-4, and ADAMTS-5 levels were higher in the TBHP-only group than in the control group. Similar to treatment with the autophagy activator Rap, pretreatment with CUR inhibited these TBHP-induced alterations, whereas CQ, 3-MA, and compound C all alleviated the inhibitory effect of CUR (Figures 9(a)–9(g)). Furthermore, CUR treatment ameliorated TBHP-induced oxidative stress and mitochondrial impairment in human NP cells, which was reflected by a decrease in ROS production, mPTP opening, and MDA level and an increase in *ΔΨ*m and ATP content, and this amelioration was consistent with the result obtained following induction with Rap (Figures 9(h)–9(m)). However, inclusion of CQ, 3-MA, or compound C reversed the CUR-mediated amelioration (Figures 9(h)–9(m)). Overall, these results demonstrated that CUR inhibits TBHP-induced apoptosis, senescence, ECM degradation, oxidative stress, and mitochondrial dysfunction by facilitating autophagic flux.

### 3.10. CUR Ameliorates IDD Development in a Rat Model In Vivo

**Figure 10:**
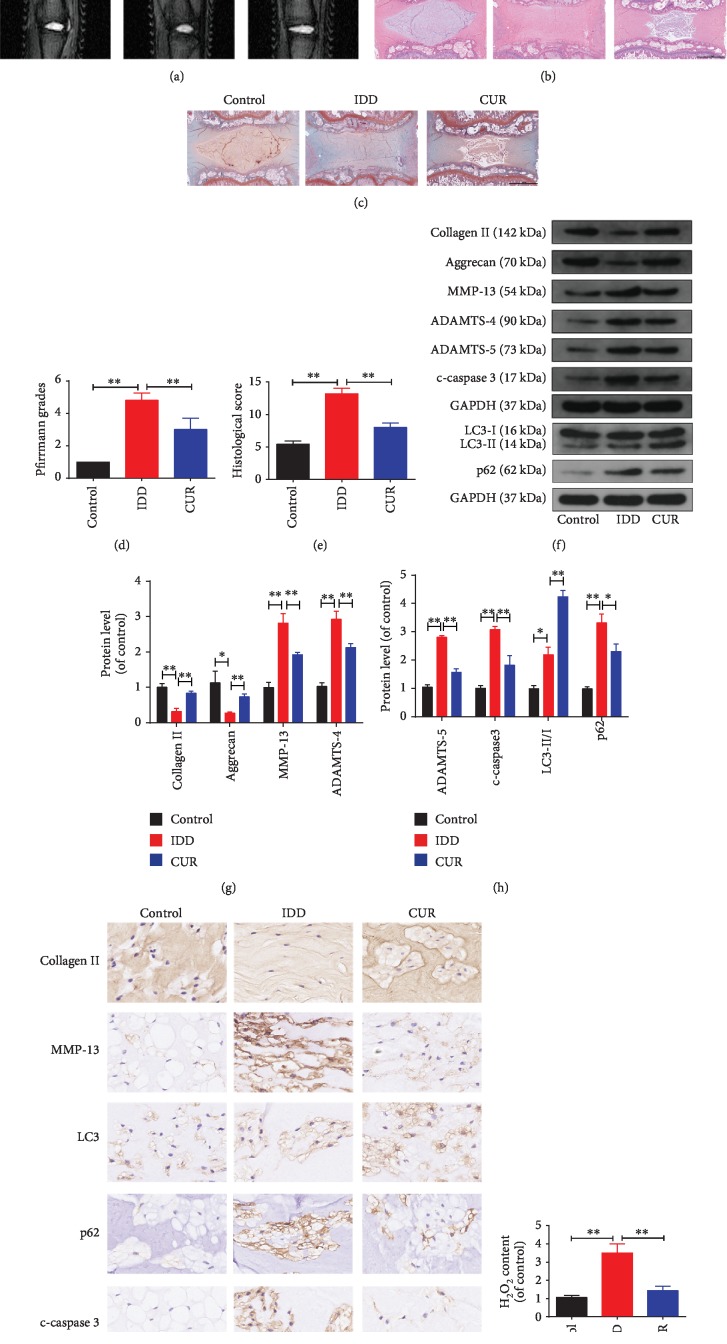
CUR treatment ameliorates rat IDD *in vivo*. (a) The discs from the tails of rats obtained from different treatment groups were examined using MRI at T2-weighted signal (white arrows). (b, c) HE and SO staining of the whole rat tail disc. Scale bar: 1 mm. (d) Quantitative analysis of the degree of disc degeneration based on the Pfirrmann grade system using MRI images. (e) The histological scores of the tail discs according to the histological grading scale. (f–h) The levels of type II collagen, aggrecan, MMP-13, ADAMTS-4, ADAMTS-5, LC3, p62, and cleaved caspase-3 proteins in the disc samples obtained from the rat models were measured by western blotting. (i) Immunohistochemical staining showing the expression of type II collagen, MMP-13, LC3, p62, and cleaved-caspase3 proteins in the disc samples of the rat model. (j) The content of H_2_O_2_ in the disc samples. Scale bar: 40 *μ*m. GAPDH was used as an internal control. Data are represented as the mean ± SD. ^∗∗^*P* < 0.01, ^∗^*P* < 0.05, *n* = 6.

Considering all the experimental results obtained *in vitro*, we constructed a needle puncture-induced IDD model in rats to investigate the therapeutic effect of CUR on IDD *in vivo*. The level of IDD was measured using MRI and was evaluated based on Pfirrmann MRI grade scores. The MRI results showed that T2-weighted signal intensities were higher in the CUR treatment group than in the IDD group (Figure 10(a)), whereas the Pfirrmann MRI grade scores were lower in the CUR group than in the IDD group (Figure 10(d)). Next, we assessed the histomorphological changes in the IVD tissues in the rat model by using HE and SO staining (Figures 10(b) and 10(c)): In the control group, the oval-shaped NP constituted a large volume of the disc in the midsagittal cross-section, as confirmed by HE staining, and the NP area contained a large amount of glycosaminoglycan, as indicated by strong SO staining. The star-shaped cells in the NP area were evenly distributed and surrounded by an abundant ECM. Relative to the control group, the IDD group showed a collapse of the disc height together with notable fibrous-tissue invasion. After CUR treatment, the loss of NP tissue and the destruction of disc structure were alleviated as compared with the effects observed in the IDD group. The histological score also indicated the CUR protection against IDD development (Figure 10(e)). Western blotting results showed that CUR treatment increased the levels of type II collagen and aggrecan and the ratio of LC3-II/I and decreased the levels of cleaved caspase-3, MMP-13, ADAMTS-4, ADAMTS-5, and p62 compared to the IDD group (Figures 10(f)–10(h)), and immunohistochemical staining results for type II collagen, MMP-13, cleaved caspase-3, LC3, and p62 were consistent with western blotting results (Figure 10(i)). Lastly, compared with the IDD group, there was a significant decrease in H_2_O_2_ content in the CUR treatment group (Figure 10(j)). Together, these findings suggest that CUR ameliorates the progression of IDD *in vivo*.

## 4. Discussion

A global review of LBP prevalence in the adult general population showed a point prevalence of 12%–33% and 1-year prevalence of 22%–65% [33]. Although LBP early diagnosis and prevention have been improved as a result of extensive research focused on the mechanism of IDD, the current clinical treatment is limited to symptom relief and does not effectively reverse the pathology of IDD, which eventually leads to recurrences of disc disease or loss of disc function [34–36]. Therefore, it is of paramount importance to identify efficacious drugs that prevent IDD progression by acting on its pathological mechanisms. This study found that CUR can enhance autophagy and restore autophagic flux and thereby reduce ROS in human NP cells and, consequently, inhibit mitochondrial dysfunction and excessive NP cell apoptosis, senescence, and ECM degradation induced by oxidative stress. This *in vivo* study further demonstrates the therapeutic effects of CUR on IDD.

ROS constitute a type of unstable and highly reactive molecules produced by aerobic metabolism and include the O_2_^–^, H_2_O_2_, and hydroxyl radical (^·^OH). Accumulating evidence indicates that degenerated IVDs exhibit an increased level of ROS, which supports the *in vitro* TBHP-induced model used in this study [37]. Mitochondria represent the main target of ROS attack, and dysfunctional mitochondria are also the main site of excessive ROS generation. This creates a vicious cycle that results in a sustained ROS production that leads to oxidative stress and ultimately cell death [38], which contributes substantially to the pathogenesis of IDD. We investigated the effect of CUR on oxidative stress and mitochondrial dysfunction induced by TBHP. As expected, CUR pretreatment attenuated the intracellular accumulation of ROS and MDA, prevented the prolonged mPTP opening, and alleviated the impairment of *ΔΨ*m, ATP production, and SOD activity in human NP cells stimulated with TBHP. Dysfunctional mitochondria can release proapoptotic proteins to form the apoptosome and to activate caspase cascades for NP cell apoptosis. A decrease in antiapoptotic Bcl-2 levels, an increase in proapoptotic Bax levels, and the translocation of cytochrome c from mitochondria to the cytoplasm are reported to be characteristics of NP cell apoptosis through the mitochondrial pathway [27]. We found that the level of apoptosis in NP cells was drastically increased when TBHP was applied and that CUR alleviated the TBHP-induced apoptosis through the mitochondrial pathway. Senescence also plays a key role in the occurrence and development of IDD. With aging, NP cellular senescence appears naturally, but this process can be accelerated by several pathological events, including oxidative stress [39]. Previous work demonstrated that in a degenerative IVD, NP cellular senescence accumulates and is associated with reduced viability, enhanced catabolic metabolism, increased inflammatory response, and compromised self-repair [40]. We found that CUR markedly decreased both SA-*β*-gal activity and p16 levels, which suggests that CUR potently inhibits oxidative stress-induced human NP cell senescence, a finding that agrees with the attenuation of IDD development. The IVD physiological function depends on the molecular composition of the NP ECM, which mainly consists of type II collagen and proteoglycans. Type II collagen provides tensile strength to the disc [41], and aggrecan—the most common type of proteoglycan—functions to enable the NP imbibe water and aids in nutrient diffusion from the periphery by maintaining an osmotic gradient [42]. A progressive decrease in type II collagen and aggrecan content, which arises due to an imbalance between matrix anabolism and catabolism, is the main pathological feature of IDD [9]. Our study confirmed that CUR corrects the ECM synthesis/degradation imbalance and retains the matrix components by promoting type II collagen and aggrecan expression and inhibiting the expression of the ECM catabolic enzymes MMP-3, MMP-13, ADAMTS-4, and ADAMTS-5; this indicates the beneficial effect of CUR on NP ECM homeostasis under oxidative stress. Furthermore, the results of our *in vivo* study showed that CUR treatment ameliorated the needle puncture-induced degeneration of the rat tail disc, which further confirmed the aforementioned results of *in vitro* experiments.

Autophagy, which means “self-eating,” plays a critical role in maintaining cellular homeostasis by degrading and recycling intracellular damaged organelles and proteins in response to increased metabolic requirements of cells or to environmental stressors such as starvation, hypoxia, and oxidative stress [43]. Nondegenerative rat NP and AF cells have been reported to exhibit a low basal level of autophagy, and this autophagy activity is markedly increased in degenerative rat NP and AF cells [44, 45]. However, Jiang et al. obtained an opposite result: Beclin-1 expression and LC3-II/I ratio were lower in the NP cells of patients with IDD than in the NP cells of patients with lumbar vertebral fracture [46]. Thus, the upregulation or downregulation of autophagy activity during IDD might be related to the functional diversity of autophagy. Moreover, a putative role of autophagy in the pathogenesis of IDD has been suggested in other previous studies [47]. For example, Chen et al. reported that activating autophagy can suppress the expression of MMP-3, MMP-13, ADAMTS-4, and ADAMTS-5 in human NP cells treated with interleukin-1*β* but that inhibiting autophagy produces the opposite effect [48]. Miyazaki et al. showed that recombinant human SIRT1 protects against nutrient deprivation-induced mitochondrial apoptosis through autophagy induction in human NP cells [49]. Collectively, these results showed that by adjusting autophagy activity, the degenerative phenotype of NP cells induced by various pathological factors can be substantially mitigated; this suggests that autophagy in NP cells might represent an effective target for the treatment of IDD.

CUR is a polyphenol that has been confirmed to exert multiple pharmacological effects in both *in vitro* and *in vivo* models [50, 51]. Intriguingly, CUR has been reported to either promote or inhibit autophagy activity in various cells [52–54]. In this study, we found that CUR induced autophagy and enhanced autophagic flux in human NP cells, and we further studied the potential molecular mechanisms by which CUR promotes autophagy. We first focused on the AMPK/mTOR/ULK1 signaling pathway because this pathway is critical for the regulation of autophagy activation: AMPK, a key energy sensor, can directly phosphorylate ULK1 (the mammalian homolog of yeast ATG1), which, in turn, initiates autophagy [55]; conversely, mTOR, an evolutionarily highly conserved serine/threonine protein kinase, was identified to function as a negative regulator of autophagy by suppressing ULK1, and this mTOR action can be countered by AMPK signaling [56, 57]. CUR has been reported to modulate autophagy by inhibiting mTOR [58] or activating the AMPK pathway [59]. Accordingly, our study revealed the involvement of the AMPK/mTOR/ULK1 pathway in CUR-mediated autophagy activation.

The accumulation of intracellular pathogenic wastes due to autophagy disorders has been implicated in the occurrence and development of various diseases, including IDD [43, 47]. During the complete execution of the autophagy process, the fusion of autophagosomes and lysosomes depends on the membrane proteins of the lysosome, and the subsequent degradation of the trapped cargo depends on the activity of lysosomal acidic hydrolases; this indicates that the lysosome is essential for autophagy and that lysosomal dysfunction might result in damage of autophagic flux and disturbance of cellular homeostasis. Recently, Chi et al. showed that the pathological mechanism by which mutations in the LAMP2 gene lead to Danon disease is related to the impairment of autophagic flux and the excessive accumulation of autophagosomes [60]. Moreover, Li et al. revealed a critical role of lysosomal dysfunction caused by inhibition of lysosomal hydrolase activity in the pathogenesis of cadmium-induced neurotoxicity [29]. In this study, we found that autophagic flux was perturbed during the fusion between autophagosomes and lysosomes as well as during the degradation of autophagic cargo in human NP cells under oxidative stress. Notably, CUR pretreatment repaired lysosomal function and autophagosome-lysosome fusion and thus eventually rescued the impairment of autophagic flux by oxidative stress.

We further investigated the role of autophagic flux in the observed cytoprotective effect of CUR in human NP cells stimulated with TBHP. The beneficial effects of CUR were found to be consistent with those produced by Rap, which is known to inhibit mTOR and thus promote autophagy activity. Subsequently, we used 3-MA, CQ, and compound C to suppress autophagy activity in human NP cells: 3-MA is a selective PI3K inhibitor that mainly hinders the initial stage of autophagy; CQ blocks lysosomal acidification and thereby interferes with a key step in autophagic flux; and compound C is an extensively used specific inhibitor of AMPK. Our results demonstrated that both inhibition of autophagy initiation and impairment of autophagic flux diminished the ability of CUR to protect human NP cells against TBHP-induced oxidative stress, apoptosis, senescence, and ECM degradation. Collectively, the findings of this study suggest that the protective effects of CUR on TBHP-treated human NP cells can be attributed to the enhancement of autophagy and the restoration of autophagic flux.

NP cell apoptosis and senescence and the imbalance between matrix anabolism and catabolism are widely recognized as the main contributors to IDD [5]. Concurrently, abundant evidence indicates that oxidative stress potently triggers these pathological processes [6]. In this study, we used TBHP to induce excessive ROS production, which resulted in oxidative damage to intracellular macromolecules and mitochondria. Our data further showed that autophagic degradation was obstructed in TBHP-treated human NP cells. Undergraded autophagosomes containing cargo and damaged mitochondria can induce increased ROS production and thus create a positive-feedback loop that leads to progressive cellular injury [61, 62]. Clearing accumulated autophagosomes and damaged mitochondria through restoration of impaired autophagic flux would help retain ROS at normal levels and stabilize mitochondrial function, which would ultimately maintain cellular homeostasis. Therefore, in this study, the enhancement of autophagy and the restoration of autophagic flux by CUR might have indirectly played a protective role by scavenging excessive ROS through the aforementioned molecular mechanisms.

CUR exerts antioxidant effects by increasing the activities of antioxidant enzymes such as SOD [63]. In this study, our results showed that CUR pretreatment reversed the downregulation of SOD activity induced by TBHP. Moreover, the ROS production was partially restored after cotreatment with autophagy inhibitor and CUR. Therefore, these results suggest that the protective effects of CUR on NP cells were at least partially associated with its regulation of SOD activity.

A few limitations of this study are the following. First, the rat IVD experiences different biomechanical characteristics compared with that of human. More studies using a monkey, goat, or dog may provide further insights. Second, as CUR is a Chinese patent medicine that has many pharmacological effects, the protective effect of CUR on NP cells may be not only through regulating autophagy activity but also through other signaling pathways. Thus, more investigations in the future are needed to specifically investigate the protective effect of autophagic flux restoration in the process of IDD.

## 5. Conclusion

This study demonstrated that CUR treatment induced autophagy and enhanced autophagic flux through the AMPK/mTOR/ULK1 signaling pathway, which protected human NP cells against oxidative stress-triggered apoptosis, senescence, and ECM degradation by alleviating excessive ROS production and mitochondrial dysfunction (Supplementary Fig. [Supplementary-material supplementary-material-1]). These effects were limited upon inhibition of autophagy initiation or obstruction of autophagic flux in NP cells. Furthermore, the results of *in vivo* analysis demonstrated the therapeutic effect of CUR on IDD. Our findings suggest that CUR may be a potential therapeutic agent for IDD treatment.

## Figures and Tables

**Table 1:** Sequences of primers used for qRT-PCR.

Gene	Oligonucleotide sequence		Product size (bp)
	Forward (5′–3′)	Reverse (5′–3′)	
Type II collagen	AGAACTGGTGGAGCAGCAAGA	AGCAGGCGTAGGAAGGTCAT	142
Aggrecan	TGAGCGGCAGCACTTTGAC	TGAGTACAGGAGGCTTGAGG	287
MMP3	TTCCTTGGATTGGAGGTGAC	AGCCTGGAGAATGTGAGTGG	248
MMP13	CCCAACCCTAAACATCCAA	AAACAGCTCCGCATCAACC	147
ADAMTS4	ACCCAAGCATCCGCAATC	TGCCCACATCAGCCATAC	246
ADAMTS5	GACAGTTCAAAGCCAAAGACC	TTTCCTTCGTGGCAGAGT	204
GAPDH	TCAAGAAGGTGGTGAAGCAGG	TCAAAGGTGGAGGAGTGGGT	115

## Data Availability

The data used to support the findings of this study are included within the article.

## Abbreviations

LBP: Lower back pain
IVD: Intervertebral disc
IDD: Intervertebral disc degeneration
CUR: Curcumin
NP: Nucleus pulposus
ECM: Extracellular matrix
AF: Annulus fibrosus
TBHP: *tert*-Butyl hydroperoxide
ROS: Reactive oxygen species
mPTP: Mitochondrial permeability transition pore
CQ: Chloroquine
3-MA: 3-Methyladenine
CTSB/CTSD: Cathepsin B/D
DHE: Dihydroethidium
MDA: Malondialdehyde
Rap: Rapamycin
SA-*β*-gal: Senescence-associated *β*-galactosidase
SOD: Superoxide dismutase.

## Supplementary Materials

Supplementary Materials The mechanism of curcumin-induced autophagy activation and autophagic flux restoration in alleviating TBHP-induced oxidative damage and mitochondrial dysfunction in human NP cells.
