# Supplementary figures and images for: Restoration of Autophagic Flux Rescues Oxidative Damage and Mitochondrial Dysfunction to Protect against Intervertebral Disc Degeneration
Source: Oxid Med Cell Longev. 2019 Dec 30;2019:7810320. doi: 10.1155/2019/7810320 (PMC6954474; doi:10.1155/2019/7810320)

## Graphical Abstract

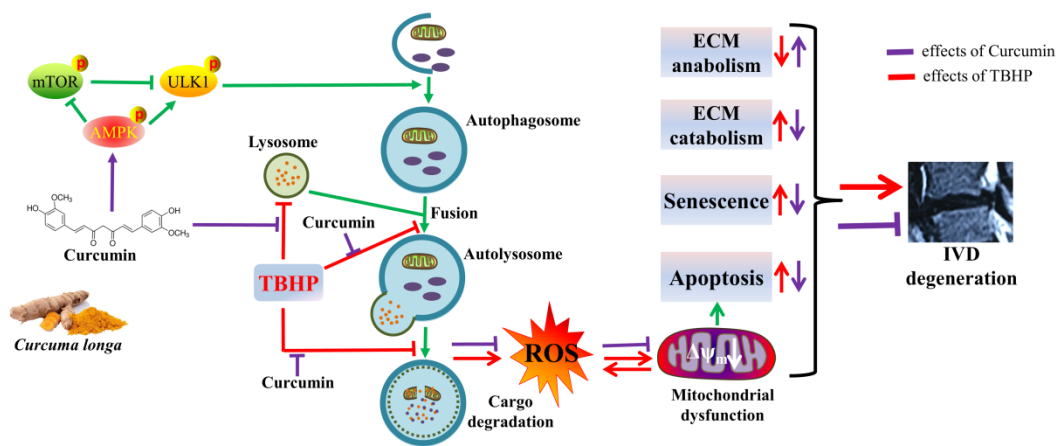

Supplement: Supplementary Materials — The mechanism of curcumin-induced autophagy activation and autophagic flux restoration in alleviating TBHP-induced oxidative damage and mitochondrial dysfunction in human NP cells. [file 7810320.f1.pdf]
